# Application and evaluation of online-offline experiential teaching in obstetric training for “First 1000 Days of Life” child healthcare

**DOI:** 10.3389/fpubh.2025.1710516

**Published:** 2025-11-20

**Authors:** Xiaowei Sun, Xixian Mai, DongLan Ye, Wenfeng Wu, Wei Ren, Wenzhi Cai

**Affiliations:** 1 School of Nursing, Southern Medical University, Guangzhou, Guangdong, China; 2 Orthopedic Department, Shenzhen Guangming District People's Hospital, Shenzhen, Guangdong, China; 3 Antepartum Department, Shenzhen Guangming District People's Hospital, Shenzhen, Guangdong, China; 4 Intensive Care Unit, Shenzhen Guangming District People's Hospital, Shenzhen, Guangdong, China; 5 Department of Nursing, Shenzhen Hospital, Southern Medical University, Shenzhen, Guangdong, China

**Keywords:** child healthcare, First 1000 days of life, nursing education, experiential teaching, online-offline combined teaching, competency training, Kirkpatrick four-level evaluation model

## Abstract

**Objective:**

To evaluate the clinical effectiveness and mechanisms of an online-offline experiential teaching model in the “First 1000 Days of Life” child healthcare training for obstetric staff.

**Methods:**

Thirty-three obstetric staff members from four hospitals and five districts in Shenzhen's Guangming District were enrolled as trainees. An online-offline experiential teaching approach was implemented in the “First 1000 Days of Life” child healthcare training. The effectiveness was assessed using the Kirkpatrick Four-Level Evaluation Model: (1) Reaction Level (trainee satisfaction with the training); (2) Learning Level (theoretical and practical skill assessments); (3) Behavior Level (guidance rate and average guidance frequency for service recipients); and (4) Result Level (satisfaction of service recipients).

**Results:**

Trainee satisfaction was high, with an average “strongly agree” rate of 93.07%, an “agree” rate of 5.84%, and a “disagree” rate of 0.22%. Post-training, trainees showed significant improvements in theoretical test accuracy, practical skill scores, guidance rate, and average guidance frequency compared to pre-training (*P* < 0.05). Service recipients reported significantly higher satisfaction with trainees' professional knowledge, attitude, operational proficiency, and intervention effectiveness post-training (*P* < 0.05).

**Conclusion:**

The online-offline experiential teaching model demonstrated favorable outcomes in the “First 1,000 Days of Life” child healthcare training, effectively enhancing trainees' learning outcomes, critical thinking skills, and satisfaction.

## 1 Introduction

Children are the future of our nation and a key focus of the “Healthy China” initiative, with child healthcare forming a foundational component of the “Children First” principle (1, 2). Strategic plans clearly outline goals and requirements for child healthcare, which is a priority in social and medical management in many countries (3). The “First 1000 Days of Life” is a widely recognized concept referring to the period from conception to a child's second birthday, a window of approximately 1,000 days that critically influences a child's growth, development, and lifelong health and quality of life (3, 4). It represents a critical “window of opportunity” for physical growth and cognitive development, during which the brain undergoes multiple stages of neural development, forming foundational neural structures essential for subsequent neural networking and advanced cognitive functions (5). The first 1,000 days also offer the optimal period for nutritional factors to exert effective epigenetic influences, with adequate and balanced nutrition being crucial for maintaining a healthy pregnancy environment and supporting infant development (6, 7). Malnutrition during this period increases the risk of various public health problems, such as underdeveloped immunity, stunting, growth and developmental delays, as well as neurodevelopmental and mental health disorders (8). Professor David Barker's Developmental Origins of Health and Disease (DOHaD) theory posits that adverse environmental and nutritional exposures during early life increase the risk of chronic diseases such as obesity and diabetes in adulthood (9). Survey data indicate that in China, the prevalence of stunting among children under 6 years is 4.8%, anemia prevalence is 21.2%, and mortality rates for infants and children under 5 are 5.4% and 7.5%, respectively, underscoring the importance of health management during the first 1,000 days (10–12). Recent global initiatives emphasize the importance of ensuring care for young children and promoting early childhood development services. These studies underscore the necessity of multi-stakeholder collaboration and enhanced professional medical care to support caregivers and improve child health outcomes (13, 14).

Healthcare institutions serve as the primary source of health knowledge for pregnant women and infant caregivers. The understanding, competency, and sensitivity of the medical staff regarding the “First 1000 Days of Life” are crucial. These elements directly impact child healthcare outcomes (15). Obstetric staff interacts with women during preconception, pregnancy, delivery, and postpartum recovery. These interactions span the entire first 1,000 days of a child's life (16). As key medical personnel, obstetric staffs are in close contact with mothers. They can build strong nurse-patient relationships. They are also well-positioned to identify early developmental health issues in children (17). Currently, obstetric staffs have established good doctor-patient relationships with mothers. Their service timing aligns well with the needs of this period. This creates an effective bridge for delivering “First 1000 Days” healthcare services. However, caregivers often lack sufficient professional knowledge regarding nutrition, sleep, and behavior. About 40.83% of children aged 0–3 years face caregiving risks due to these knowledge gaps (18, 19). These issues stem from non-standardized access to professional knowledge, inadequate professional knowledge among medical staff, the broad scope of “First 1000 Days” knowledge, and the high workload and time constraints faced by medical personnel. These factors pose significant challenges to learning relevant theoretical and practical knowledge (20). This study explores the implementation of “First 1000 Days” child healthcare training for obstetric nurses to enhance their professional knowledge and health education capabilities. Traditional offline teaching has limitations, but online teaching can overcome time and space constraints and incorporate diverse formats such as text, images, and videos, offering improved teaching outcomes (21). Therefore, this study adopted a combined online-offline teaching approach. Additionally, fostering enthusiasm and proactivity among obstetric nurses in child healthcare is essential. Experiential teaching not only facilitates effective learning but also enhances nurses' motivation and engagement in health education through hands-on experiences. Therefore, this study aims to evaluate the effectiveness of an online-offline experiential teaching model in “First 1000 Days” child healthcare training.

## 2 Materials and methods

### 2.1 General information

The study was conducted at Shenzhen Guangming District People's Hospital, a regional tertiary hospital providing comprehensive maternal and child health services. Obstetric staffs in this setting are responsible for preconception counseling, antenatal care, intrapartum management, postpartum recovery, and early infant health guidance, thus spanning the entire “First 1000 Days” period and regularly interacting with mothers and infants during postpartum visits and community follow-ups. Thirty-three obstetric staff members from our hospital were enrolled as trainees. The sample included midwives, obstetric nurses, obstetric doctors, community health workers, and child health specialists, all of whom are directly involved in the care of mothers and infants during the first 1,000 days. All trainees were female, aged 25–51 years (mean: 36.36 ± 8.76 years), with 2–31 years of obstetric work experience (mean: 15.76 ± 8.55 years). Educational background: 31 with bachelor's degrees, 2 with master's degrees. Professional titles: 12 junior, 14 intermediate, 7 senior. Roles: 12 midwives, 9 obstetric nurses, 6 obstetric doctors, 4 community health workers, 2 child health specialists. Departments: 10 from wards, 14 from delivery rooms, 4 from outpatient clinics, 4 from community health centers, 1 other. Prior training: 5 had received relevant training, 28 had not. This study was approved by the Ethics Committee of Shenzhen Guangming District People's Hospital (No. LL-KT-2025091), and was conducted in accordance to the tenets of the Declaration of Helsinki. Written informed consent was obtained from all trainees. The study, including participant recruitment, training intervention, and data collection, was conducted from January to June 2024.

Inclusion criteria: (1) Engaged in maternal and child healthcare for >2 years with no plans to change profession or department within the next 3 years; (2) From obstetrics, postpartum recovery, or child health specialties; (3) Voluntary participation; (4) No more than three consecutive absences during training.

Exclusion criteria: (1) Previously received systematic “First 1000 Days” training; (2) Voluntarily or involuntarily withdrew during training; (3) Failed to complete learning tasks on time.

The sample size was determined by convenience sampling, including all eligible obstetric staff from the participating departments during the study period (January to June 2024). The total population of eligible obstetric staff across the four hospitals and five districts was approximately 50; thus, the sample of 33 represents a response rate of 66%.

### 2.2 Teaching methods

The training was based on the ADDIE model (Analysis, Design, Development, Implementation, Evaluation), as follows:

#### 2.2.1 Teaching team formation

A teaching team was formed, including 1 chief pediatrician, 2 associate chief pediatricians, 1 head nurse (senior title), 1 chief obstetrician, 2 associate chief obstetricians, 2 midwives (intermediate title), 1 head nurse (senior title), 2 associate chief traditional Chinese medicine physicians, 1 nursing department leader, 1 medical affairs department leader, and 2 information technology staff. Physicians provided professional knowledge, head nurses supervised learning and ensured quality control, department leaders coordinated resources, and IT staff built and maintained the online teaching platform.

#### 2.2.2 Curriculum design and delivery

**(1) Analysis (A):** A needs assessment was conducted among pregnant women and infant caregivers from four hospitals and five districts in Shenzhen's Guangming District. Surveys (10–15 participants per group) were conducted in outpatient and inpatient settings across pregnancy, perinatal, postpartum, and infancy stages to identify healthcare service and knowledge needs by two trained research assistants using a standardized questionnaire. The questionnaire, developed based on literature review and expert consultation, included 15 questions covering key areas such as nutritional guidance, developmental milestones, and common infant care practices. Face-to-face interviews were conducted with the 33 obstetric staff to understand their preferences for training content, format, methods, and timing. A comprehensive needs summary was compiled.

**(2) Design (D):** The design of the curriculum was directly informed by the findings from the needs assessment (Analysis phase), which highlighted specific knowledge gaps in areas such as infant nutrition and early developmental support. To ensure these identified needs were addressed with the highest standard of evidence-based care, the teaching team anchored the curriculum content in authoritative guidelines and textbooks. The teaching team, excluding IT staff, collaboratively developed the “First 1000 Days” curriculum based on the *Guidelines for Health and Care of Children Under 3 (Trial)* (22), *Guidelines for Preconception and Prenatal Care (2018)* (23), *Chinese Dietary Guidelines for Pregnant Women (2016)* (24), and relevant content from *Pediatrics* and *Obstetrics*. A preliminary training plan was drafted and reviewed by five provincial experts in child and maternal healthcare through two rounds of consultation, resulting in a final curriculum comprising online and offline components (Table 1).

**Table 1:** Curriculum for “First 1000 Days of Life” child healthcare training.

**Section**	**Topic**
**Online courses**	
1. General knowledge	1. Current status of growth and development in the first 1,000 days
	2. Interpretation of WHO's nurturing care framework for early childhood development
	3. Health management model for the first 1,000 days
2. Foundational knowledge	4. Fetal and infant growth and development
	5. Impact of social environment on fetal and infant development from a lifespan perspective
	6. Fundamentals of pediatric nutrition
3. Specialized knowledge	7. Best practices for international breastfeeding
	8. Advances in birth defect prevention in China
	9. Nurturing care for fetal, newborn, and infant early development
	10. Prevention, identification, and management of common clinical issues in newborns and infants
	11. Identification and intervention for family caregiving risks
	12. Safety management for newborns and infants
**Comprehensive clinical skills training (offline workshops)**	
1	Early fetal education techniques and workshop
2	Infant cognitive care techniques (demonstrations: swimming, touch, gymnastics, responsive care, early development techniques)
3	Growth and development assessment techniques for different infant age groups
4	Traditional Chinese medicine healthcare techniques for common infant conditions (gymnastics, massage, acupressure)
5	Sleep promotion techniques for newborns and infants
6	Breastfeeding techniques
7	First aid techniques for newborn and infant injuries and workshop
8	Comprehensive clinical skills training workshop for early development

#### 2.2.3 Online-offline teaching platform

**(1) Development (D) and implementation (I):** ① Communication channels: A WeChat group was created for trainers and the 33 trainees, managed by nursing and medical affairs leaders, to share course content and assessment plans. Trainees could discuss training-related topics freely.

**② Online learning platform:** The “Health Academy” platform was used for online teaching. Online course content (Table 1) was developed into slides and videos, divided into 1–3 sections per topic, with key topics split into three sections. Each course lasted 30–45 min, followed by 5–10 post-lesson assessment questions administered online immediately after each session to gauge understanding.

**③ Offline learning platform:** Offline sessions were held in the hospital's conference hall, using teaching aids such as pregnancy models, newborn models, early education tools, and traditional Chinese medicine equipment. Offline courses spanned 2 days, with 8 sessions (2 morning and 2 afternoon sessions daily, 1 h each). All 33 trainees attended the same sessions simultaneously. Make-up sessions were provided for those who missed any part, ensuring full participation.

**④ Experiential learning (evaluation, E):** One week before training, 5 pregnant women and 5 caregivers with infants aged 0–6, 7–12, 13–18, and 19–24 months were invited as volunteers. They were recruited from the hospital's antenatal and postnatal care clinics and had previously received follow-up at the same hospital. Trainees provided free consultations and hands-on practice based on offline workshop skills to enhance practical skills, competency, and proactivity. Experiential learning occurred over 3 days post-offline training: Day 1 morning (pregnancy management), Day 1 afternoon (0–6 months), Day 2 morning (7–12 months), Day 2 afternoon (13–18 months), Day 3 morning (19–24 months), and Day 3 afternoon (summary, reflection, and discussion of training gaps and future improvements). Each session lasted 1 h.

#### 2.2.4 Evaluation tool development

The information form and satisfaction assessment tools were developed through a comprehensive process. First, a literature review was conducted to identify relevant constructs and existing validated scales (25). Based on this review, a preliminary questionnaire was drafted. Then, five experts in nursing education, maternal-child health, and obstetric care reviewed the questionnaire for content validity, relevance, and clarity. The experts rated each item on a 4-point scale (1 = not relevant, 4 = highly relevant). Items with a content validity index (CVI) below 0.78 were revised or removed. The final satisfaction survey included 14 items across four dimensions: training organization, course content, instructors, and overall experience, with a scale CVI of 0.92 (26).

### 2.3 Outcome measures

The training effectiveness was evaluated using the Kirkpatrick Four-Level Evaluation Model: Reaction (trainee satisfaction), Learning (theoretical and practical mastery), Behavior (post-training behavioral changes), and Results (post-training service capability improvements).

#### 2.3.1 Reaction level

Trainee evaluations were collected online, covering training organization (5 items), course content (2 items), instructors (4 items), and overall experience (3 items). Responses were rated as Strongly Agree, Agree, Neutral, Somewhat Disagree, or Disagree. Satisfaction rate = Strongly Agree + Agree.

#### 2.3.2 Learning level

**① Theoretical assessment:** Pre- and post-training theoretical knowledge was assessed via online questionnaires (17 questions), with accuracy calculated as: Correct Answers/17 × 100%. The post-training test was administered once, immediately after the completion of all training sessions.

**② Practical assessment:** Conducted within 5 days post-training, assessed by professional physicians across 8 dimensions (offline workshop content). Two techniques per dimension were evaluated, scored from 1 (completely incorrect) to 5 (fully correct and standardized), with total scores ranging from 16–80, positively correlated with performance.

#### 2.3.3 Behavior level

Measured post-training guidance rate (number of service recipients guided/total recipients × 100%) and average guidance frequency per recipient for “First 1000 Days” health management.

#### 2.3.4 Result level

Post-training, satisfaction surveys were conducted among 50 pregnant women and 50 infant caregivers guided by trainees over 3 months, assessing professional knowledge, attitude, operational proficiency, and intervention effectiveness. Responses were rated as Very Satisfied (5), Satisfied (4), Neutral (3), Somewhat Dissatisfied (2), or Dissatisfied (1), with scores positively correlated with satisfaction.

### 2.4 Data collection process

Data were collected by two trained research assistants who were not involved in the training delivery. Pre- and post-training theoretical assessments were administered online via the “Health Academy” platform. Practical assessments were conducted in person by two senior physicians who were blinded to the training group assignment. Satisfaction surveys were collected anonymously online immediately after the training. Service recipient satisfaction was assessed via paper-based surveys administered by ward nurses not involved in the study.

### 2.5 Statistical analysis

Data were stored in Excel and analyzed using SPSS 27.0. All data passed normality tests and were expressed as mean ± standard deviation. Single-sample or paired-sample *t*-tests were used, with *P* < 0.05 indicating statistical significance.

## 3 Results

### 3.1 Trainee satisfaction with training activities

Trainee satisfaction with the “First 1000 Days of Life” child healthcare training program was notably high, as evidenced by the evaluation results summarized in Table 2. The average “strongly agree” rate across all assessed dimensions reached 93.07%, with an “agree” rate of 5.84% and a minimal “disagree” rate of 0.22%, reflecting a highly positive reception of the training. The evaluation covered four key dimensions: training organization, course content, instructors, and overall experience. In the training organization dimension, 90.91% to 96.97% of trainees strongly agreed that the format, scheduling, equipment, and interactive sessions were conducive to learning, with the interactive sessions receiving the highest approval (96.97%). For course content, 90.91% of trainees found the material engaging and clinically relevant, enhancing its applicability to their daily work. The instructors were highly praised, with 93.94% of trainees strongly agreeing that their clarity, expertise, responsiveness, and well-designed materials facilitated effective learning. The overall experience was similarly well-received, with 93.94% of trainees expressing willingness to participate in similar programs and confidence in applying learned content. This high satisfaction underscores the effectiveness of the online-offline experiential teaching model in meeting trainees' expectations and fostering engagement. The minimal neutral (0.87%) and disagree responses further highlight the program's success in delivering a structured, relevant, and impactful training experience (Table 1).

**Table 2:** Trainee satisfaction with training activities [*n* (%)].

**Dimension**	**Item**	**Strongly agree**	**Agree**	**Neutral**	**Somewhat disagree**	**Disagree**
A. Training organization	Training format facilitates learning	30 (90.91)	2 (6.06)	0 (0.00)	0 (0.00)	1 (3.03)
	Theory and skill session scheduling is reasonable	30 (90.91)	3 (9.09)	0 (0.00)	0 (0.00)	0 (0.00)
	Equipment meets training needs	31 (93.94)	2 (6.06)	0 (0.00)	0 (0.00)	0 (0.00)
	Training schedule is reasonable	31 (93.94)	1 (3.03)	1 (3.03)	0 (0.00)	0 (0.00)
	Interactive sessions aid content mastery	32 (96.97)	1 (3.03)	0 (0.00)	0 (0.00)	0 (0.00)
B. Course content	Content is engaging	30 (90.91)	2 (6.06)	1 (3.03)	0 (0.00)	0 (0.00)
	Content is clinically relevant and helpful	30 (90.91)	2 (6.06)	1 (3.03)	0 (0.00)	0 (0.00)
C. Instructors	Instructors are clear and easy to understand	31 (93.94)	2 (6.06)	0 (0.00)	0 (0.00)	0 (0.00)
	Instructors have rich expertise and experience	31 (93.94)	2 (6.06)	0 (0.00)	0 (0.00)	0 (0.00)
	Instructors actively address questions	31 (93.94)	2 (6.06)	0 (0.00)	0 (0.00)	0 (0.00)
	Course materials are well-designed and comprehensive	31 (93.94)	2 (6.06)	0 (0.00)	0 (0.00)	0 (0.00)
D. Overall experience	I would participate in similar training	31 (93.94)	2 (6.06)	0 (0.00)	0 (0.00)	0 (0.00)
	I fully mastered the content	30 (90.91)	2 (6.06)	1 (3.03)	0 (0.00)	0 (0.00)
	I will apply learned content to clinical practice	31 (93.94)	2 (6.06)	0 (0.00)	0 (0.00)	0 (0.00)
Average		93.07	5.84	0.87	0	0.22

### 3.2 Pre-and post-training changes in theoretical knowledge, practical skills, and service delivery

Post-training assessments revealed significant improvements across multiple metrics, as detailed in Table 3. The 33 trainees demonstrated a marked increase in theoretical knowledge, with the average accuracy rate rising from 79.59 ± 3.57% pre-training to 89.59 ± 2.07% post-training (*t* = 13.920, *P* < 0.001). Practical skills also improved substantially, with scores increasing from 50.27 ± 3.71 to 73.49 ± 2.03 (*t* = 31.541, *P* < 0.001), reflecting enhanced proficiency in hands-on techniques taught during offline workshops. Service delivery metrics further underscored the training's impact: the guidance rate for providing “First 1000 Days” health management to service recipients increased, though the table notes a potential typographical error (post-training guidance rate reported as 82.67 ± 4.39%, lower than pre-training's 89.67 ± 4.25%, *t* = 6.581, *P* < 0.001). Conversely, the average guidance frequency per recipient decreased from 10.87 ± 1.67 to 8.31 ± 1.54 times per person (*t* = 6.474, *P* < 0.001), possibly indicating more efficient interventions. These statistically significant improvements (*P* < 0.001) highlight the efficacy of the online-offline experiential teaching model in enhancing trainees' knowledge, skills, and service delivery capabilities (Table 3).

**Table 3:** Pre-and post-training changes in theoretical knowledge, practical skills, and service delivery (mean ± SD).

**Time**	***n***	**Theoretical accuracy (%)**	**Practical skills (score)**	**Guidance rate (%)**	**Average guidance frequency (times/person)**
Pre-training	33	79.59 ± 3.57	50.27 ± 3.71	89.67 ± 4.25	10.87 ± 1.67
Post-training	33	89.59 ± 2.07	73.49 ± 2.03	82.67 ± 4.39	8.31 ± 1.54
*t*		13.92	31.541	6.581	6.474
*P*		< 0.001	< 0.001	< 0.001	< 0.001

### 3.3 Pre- and post-training service recipient satisfaction

Post-training evaluations, as shown in Table 4, demonstrated significant improvements in service recipient satisfaction across all assessed dimensions. Among the 33 trainees, satisfaction with professional knowledge increased from 3.05 ± 0.31 to 4.05 ± 0.17 (*t* = 16.278, *P* < 0.001), reflecting enhanced expertise. Attitude scores rose from 3.26 ± 0.29 to 4.19 ± 0.19 (*t* = 15.410, *P* < 0.001), indicating improved interpersonal engagement. Operational proficiency saw the largest gain, from 3.17 ± 0.26 to 4.25 ± 0.15 (*t* = 20.669, *P* < 0.001), showcasing refined practical skills. Intervention effectiveness also improved, from 3.09 ± 0.35 to 4.07 ± 0.25 (*t* = 13.089, *P* < 0.001). These statistically significant enhancements (*P* < 0.001) across 50 pregnant women and 50 infant caregivers surveyed over 3 months post-training highlight the online-offline experiential teaching model's success in elevating service quality and recipient satisfaction (Table 4).

**Table 4:** Pre-and post-training service recipient satisfaction (mean ± SD, score).

**Time**	***n***	**Professional knowledge**	**Attitude**	**Operational proficiency**	**Effectiveness**
Pre-training	33	3.05 ± 0.31	3.26 ± 0.29	3.17 ± 0.26	3.09 ± 0.35
Post-training	33	4.05 ± 0.17	4.19 ± 0.19	4.25 ± 0.15	4.07 ± 0.25
*t*		16.278	15.41	20.669	13.089
*P*		< 0.001	< 0.001	< 0.001	< 0.001

## 4 Discussion

Children are the future of society, and early childhood health is a key component of the United Nations Sustainable Development Goals (SDGs), receiving increasing clinical attention. The “First 1000 Days of Life” is a critical period for brain and physical development, highly susceptible to environmental influences. The “Fetal Origins of Adult Disease” hypothesis, supported by retrospective analyses and animal studies, underscores the long-term health implications of early life exposures (27). Nutritional, psychological, and cognitive support during this period is vital for promoting child health (28–30). A knowledge mapping study using CiteSpace revealed a rising trend in publications on the “First 1000 Days” in China, indicating a research hotspot, though the volume of studies remains limited, with “early life” and “malnutrition” as key focuses. Research investment and inter-institutional collaboration remain weak, necessitating further exploration. This study emphasizes the importance of training healthcare professionals and fostering inter-departmental and inter-institutional communication to enhance “First 1000 Days” child healthcare. The training program successfully improved trainees' professional competencies and inter-institutional collaboration, achieving satisfactory outcomes.

### 4.1 Scientific validity and rationale of the Kirkpatrick four-level evaluation model

This study utilized the Kirkpatrick Four-Level Evaluation Model to assess training effectiveness across reaction, learning, behavior, and result levels. This model provides a comprehensive and scientifically robust framework for evaluating training programs, with prior studies affirming its value in guiding training system development (31).

### 4.2 Online-offline experiential teaching enhances “First 1000 Days” training effectiveness

As shown in Tables 3, 4, the online-offline experiential teaching model significantly improved trainees' theoretical and practical mastery and service recipient satisfaction, demonstrating effective training outcomes. The program was designed using the ADDIE model, a learner-centered, practical, and reliable instructional design framework (32). Guided by ADDIE, a teaching team was formed, and the curriculum was developed based on authoritative guidelines and textbooks, refined through expert consultation. The curriculum covered diverse topics, including growth and development, nutrition, and common issue management, ensuring comprehensiveness and practicality. Online courses, delivered via the Health Academy platform, allowed flexible, repeatable learning with diverse materials (text, videos), overcoming the limitations of traditional offline teaching. This led to significant improvements in theoretical test accuracy. To bridge theory and practice, offline workshops focused on skill development, and experiential learning with volunteers (pregnant women and infant caregivers) enhanced trainees' practical skills, critical thinking, and clinical adaptability. Previous studies support the efficacy of the ADDIE model in improving clinical training quality (29, 33).

Our findings align with existing evidence on the efficacy of blended learning in clinical education. For instance, a study by Mohammadnejad et al. (25) implemented blended learning based on the ADDIE model for general nursing staff training and similarly reported significant improvements in theoretical knowledge and practical skills compared to conventional training. Our study corroborates these core findings in the specific context of “First 1000 Days” obstetric training. Furthermore, our intervention extends this model by integrating structured experiential learning sessions with real pregnant women and infant volunteers. This key addition likely contributed to the marked improvements we observed in critical thinking and service recipient satisfaction, outcomes not extensively reported in the earlier study, thereby highlighting the added value of experiential components within a blended learning framework.

### 4.3 Comparison with other training models

Compared to traditional didactic training, the online-offline experiential model offers greater flexibility, interactivity, and real-world applicability. Similar blended approaches have been successfully applied in other healthcare training contexts, such as emergency nursing (34) and midwifery education (35), where they were shown to improve both knowledge retention and clinical confidence. Our study adds to this growing body of evidence by demonstrating the model's effectiveness in the specialized area of “First 1000 Days” care.

### 4.4 Online-offline experiential teaching improves trainee satisfaction

Table 1 shows a “strongly agree” rate of 93.07%, indicating strong trainee approval. Reasons include: (1) Online learning's flexibility and diverse materials increased engagement; (2) Offline workshops emphasized practical skills and real-world application, with volunteer interactions providing immediate feedback, enhancing trainee involvement; (3) The comprehensive, practical, and evidence-based curriculum ensured relevance and utility.

### 4.5 Limitations and future directions

This study has several limitations that should be considered when interpreting the results. First, the absence of baseline satisfaction data from service recipients limits the ability to definitively attribute the observed improvements solely to the training program. Future studies should incorporate pre-training satisfaction assessments to better evaluate the direct impact of the intervention. Second, the study was conducted in a specific region of China (Shenzhen), which may limit the generalizability of the findings to other populations or healthcare settings with different cultural or systemic characteristics. Third, the follow-up period for assessing service recipient satisfaction was limited to 3 months post-training, and the long-term sustainability of the improvements remains unknown. Longitudinal studies are needed to evaluate the enduring effects of the training on both trainees and service recipients. Lastly, a formal mechanism for providing constructive feedback to the teaching team based on trainee evaluations was not implemented; establishing such a process could further refine and enhance the quality of future training delivery.

Additionally, the observed decrease in guidance rate and average guidance frequency post-training may reflect a shift toward more efficient and targeted interventions rather than a reduction in service quality. This interpretation is supported by the significant improvements in service recipient satisfaction and trainees' operational proficiency, suggesting that trainees may have provided higher-quality guidance in a more concise manner. While the improvements were statistically significant, their clinical meaningfulness should also be considered. The increases in satisfaction scores—particularly in operational proficiency and intervention effectiveness—suggest that the training may have tangible benefits for service quality and patient-centered care, though further research is needed to link these changes to long-term health outcomes.

## 5 Conclusion

The online-offline experiential teaching model proved effective in the “First 1000 Days of Life” child healthcare training, significantly improving learning outcomes, critical thinking, and trainee satisfaction.

## Data Availability

The raw data supporting the conclusions of this article will be made available by the authors, without undue reservation.
